# Exploring the impact of the national tender system on the use of costly drugs treating rheumatoid arthritis patients in ten rheumatology centers in Norway (2010–2019)

**DOI:** 10.1186/s12913-023-09975-7

**Published:** 2023-09-07

**Authors:** Alen Brkic, Andreas P Diamantopoulos, Mari Hoff, Espen Andre Haavardsholm, Bjørg Tilde Svanes Fevang, Lene Kristin Brekke, Liz Loli, Camilla Zettel, Gunnstein Bakland, Pawel Mielnik, Glenn Haugeberg

**Affiliations:** 1 Research Department, Sorlandet Hospital, Service box 416, Kristiansand, Norway; 2 Department of Neuromedicine and Movement Science, Faculty of Medicine and Health Sciences, NTNU, Norwegian University of Science and Technology, Trondheim, Norway; 3 Division of internal medicine, Section for Rheumatology, Akershus University Hospital, Lørenskog, 1478 Norway; 4 Department of Rheumatology, St Olavs Hospital, Trondheim University Hospital, Trondheim, Norway; 5 Center for treatment of Rheumatic and Musculoskeletal Diseases (REMEDY), Diakonhjemmet Hospital, Oslo, Norway; 6 Institute of Clinical Medicine, University of Oslo, Oslo, Norway; 7 Department of Rheumatology, Bergen Group of Epidemiology and Biomarkers in Rheumatic Disease, Haukeland University Hospital, Bergen, Norway; 8 Haugesund Hospital for Rheumatic Diseases, Haugesund, Norway; 9 Lillehammer Hospital for Rheumatic Diseases, Lillehammer, Norway; 10 Department of Rheumatology, Betanien Hospital, Skien, Norway; 11 Department of Rheumatology, University Hospital of North Norway, Tromsø, Norway; 12 Department for Neurology, Section for Rheumatology, Rheumatology and Physical Medicine, , District General Hospital of Førde, Førde, Norway; 13 Division of Rheumatology, Department of Medicine, Sorlandet Hospital, Kristiansand, Norway

**Keywords:** Pharmaceutical tendering, Biosimilars, Biologics, Subcutaneous, Intravenous

## Abstract

**Background:**

Biologic and targeted synthetic disease-modifying antirheumatic drugs (b/tsDMARDs) are highly effective in treating rheumatoid arthritis (RA), albeit high drug cost has restricted their use in many countries. As a countermeasure, Norway implemented pharmaceutical tendering as a cost-reducing strategy. The aim of this study was to assess the annual proportion of different b/tsDMARDs registered to treat RA patients under the influence of a Norwegian pharmaceutical tendering between 2010 and 2019.

**Method:**

The data is collected from ten Norwegian outpatient centers. The included patients are categorized as naïve, non-naïve, and current b/tsDMARD users. 13 individual b/tsDMARDs are assessed and compared with the tender rankings from each year. Overview of subcutaneous (sc) with per oral vs. intravenous (iv) and biosimilars vs. non-biosimilar are also described.

**Result:**

The tender-winning b/tsDMARD was the most or second most used drug in nine out of ten years for naïve users, seven for non-naïve users, and twice for current users. The average sum of the highest and second highest proportion among naïve, non-naïve, and current b/tsDMARD users were 75%, 53%, and 50% during the ten years, respectively. The tender-winning drug was iv in eight out of ten years. However, the average total proportion of sc and per oral b/tsDMARDs was about 70% for naïve b/tsDMARD users, 50% for non-naïve b/tsDMARD users, and 60% for current b/tsDMARD users. The main contributors to sc and per oral b/tsDMARD were etanercept (reference and biosimilar) and certolizumab pegol. The main contributors to iv b/tsDMARD were rituximab reference and infliximab biosimilar. Despite low-ranking offers, rituximab reference (offered as a second-line drug) often achieved a high proportion among non-naïve and current b/tsDMARD users. After the introduction of biosimilars, their average proportion was about 40%, 40%, and 20% for naïve, non-naïve, and current b/tsDMARD users, respectively.

**Conclusion:**

Based on observed data, a higher tender rank was associated with a higher proportion among naïve and non-naïve b/tsDMARD users. However, in most cases, sc b/tsDMARDs achieved a higher proportion with lower tender ranks than iv b/tsDMARDs with higher tender ranks.

**Supplementary Information:**

The online version contains supplementary material available at 10.1186/s12913-023-09975-7.

## Background

Rheumatoid arthritis (RA) is a chronic inflammatory joint disease with a reported prevalence of 0.5–1% [1]. RA causes joint stiffness and pain, fatigue, physical impairments, and reduced quality of life [2–4], which can further lead to reduced work capacity and work disability (unemployment or early retirement) [5, 6]. This may contribute both directly and indirectly to the cost of illness, financially affecting the patients and their families, the healthcare system, and society [5, 6].

Major improvements in clinical outcomes during the last twenty years can be attributed to the usage of biologic and targeted synthetic disease-modifying antirheumatic drugs (b/tsDMARDs) and treatment strategies focusing on treating RA patients into remission [7–11]. Despite today’s wide availability of b/tsDMARDs, their high cost limits their use in many countries, contributing to a worldwide discrepancy in access to care [12–14]. As a countermeasure, the Norwegian Hospital Procurement Trust (NHPT) has vigorously promoted annual national pharmaceutical tendering with the intention to lower drug costs [15, 16].

In a recently published paper, we examined the cost changes of b/tsDMARDs for RA treatment between 2010 and 2019 in Norway under the influence of this national pharmaceutical tendering [16]. In the present study, the aim was to assess the annual proportion of different b/tsDMARD used to treat RA patients under the influence of the Norwegian pharmaceutical tendering between 2010 and 2019.

## Methodology

### Data and patient collection

Data were collected using the software GoTreatIT ® Rheuma (www.diagraphit.com) (GTI) from ten BioRheuma centers with standardized patient monitoring of the minimum dataset of variables presented below. Further details on the BioRheuma project and the BioRheuma centers have been described in another paper [16]. In short, from each participating center, anonymized Excel data files were for every year sent for merging and statical analysis. Due to the anonymized data from each center for each year, the collected data was assessed cross-sectionally to describe annual trends in a descriptive format. The data were extracted from each participating center’s GTI database using two predefined queries for each year between 2010 and 2019. The first query retrieved RA patients registered with at least one visit in the evaluated year, generating the *current user dataset*. Data from the latest visit was used if multiple visits occurred during that year. The second query retrieved all patients starting annually on a b/tsDMARD for each year of the ten years, generating the *starting b/tsDMARD dataset*.

For included patients, collected data for each year encompassed demographic variables, biomarker variables, disease activity measures, and patient-reported outcome measures (PROMs). Demographic variables include patient age, sex, body mass index (BMI, kg/m2), current smoking status, and disease duration (calculated from the date of diagnosis until the latest visit at the outpatient clinic for the examined year).

Biomarker variables include rheumatoid factor (RF) and anti-cyclic citrullinated peptide (anti-CCP). Measures reflecting disease activity encompass laboratory measures (erythrocyte sedimentation rate (ESR), C-reactive protein (CRP)), the 28 swollen and tender joint count (SJC28 and TJC28), investigator global assessment (IGA) scored on a visual analog scale (VAS; 0-100 mm), and the composite 28 joint count Disease Activity Score using CRP (DAS28) [17].

The PROMs included were pain, patient global assessment (PGA), and fatigue scored on a VAS scale (0-100 mm). For each variable, the mean and average values were computed and presented separately for those treated with any b/tsDMARD as well as for TNFi-, non-TNFi-, and tsDMARD-groups.

### Treatment user categorization

**Fig. 1:**
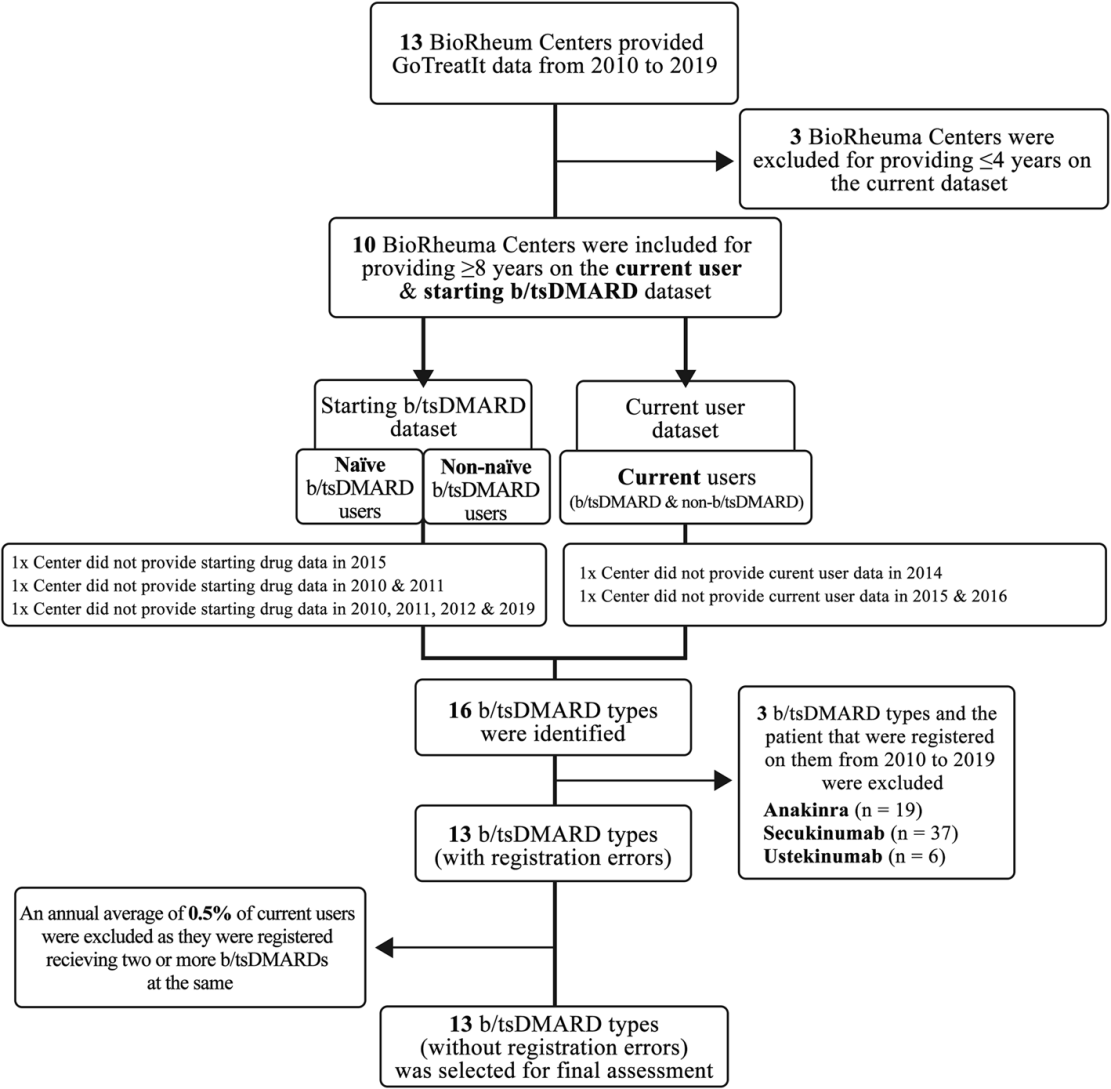
Overview of the inclusion and exclusion of BioRheuma centers and the registered b/tsDMARDs. *Abbreviation*: b/tsDMARDs = biologic and targeted synthetic disease-modifying antirheumatic drugs

The evaluated data on the treatment user groups were divided into three categories: current b/tsDMARD users collected from the *current user dataset*, and naïve and non-naïve b/tsDMARD users (both registered on a new b/tsDMARD) collected from the *starting b/tsDMARD dataset* (Fig. 1). Naïve b/tsDMARD users are those registered receiving their first b/tsDMARD, and non-naïve b/tsDMARD users are those registered receiving the given b/tsDMARD after previously being on a different b/tsDMARD. Although the *starting b/tsDMARD dataset* does not specify the sequence or treatment duration, a non-chronological drug order of previously used b/tsDMARD for each b/tsDMARD was documented.

The proportions of each b/tsDMARD, defined as the number of individual drug registrations divided by the total number of drug registrations of the given drug for a given year, were calculated and presented annually. A cross-sectional trend assessment of the proportions of each distinct group was conducted independently. All evaluated registrations were collected from the GTI system and not compared to any other prescription registry.

### BioRheuma centers selection

There were 13 BioRheuma centers initially planned for this study. However, three centers provided data (for the current dataset) for four or fewer years and were excluded from our analysis due to data deficiencies. The final current dataset consists of ten centers, each providing data for at least eight out of ten years. Details on these missing years are provided in the paper’s flowchart (Fig. 1). Unless stated otherwise, all patients were included from the included centers.

### Medication selection and analysis

A total of 16 b/tsDMARD types were identified, of which 13 were included in this study. Anakinra (*n* = 19), secukinumab (*n* = 37), and ustekinumab (*n* = 6) were excluded due to either lack of indication or very few registrations and were prescribed outside the tendering. On average, the excluded b/tsDMARDs accounted for roughly six patients (0.2% of b/tsDMARDs) each year.

The included bDMARD Tumor Necrosis Factor inhibitors (TNFi) were intravenous (iv) infliximab reference (Remicade®) [1999] (presented with the trade name in parenthesis and the year of reaching the market in brackets), iv infliximab CT-P13 (Remsima®/Inflectra®) [2013], subcutaneous (sc) etanercept reference (Enbrel®) [2000], sc etanercept SB4 (Benepali®) [2016], sc adalimumab reference (Humira®) [2003], sc golimumab (Simponi®) [2009], and sc certolizumab pegol (Cimzia®) [2009]. The bDMARD non-TNFi were iv abatacept (Orencia®) [2007, and sc from 2012], iv rituximab reference (MabThera®) [1998], iv rituximab GP2013 (Rixathon®) [2017], and iv tocilizumab (RoActemra®) [2009, and sc from 2014]. The included tsDMARDs were per oral (po) baricitinib (Olumiant®) [2017] and po tofacitinib (Xeljanz®) [2017]. The distinction between iv and sc variants of tocilizumab and abatacept was not registered in GoTreatIt. As they arrived initially to market as iv, they were labeled iv throughout this study.

Patients who received double or multiple b/tsDMARDs (Fig. 1) were assessed as registration errors due to inconsistencies with our treatment guidelines and, as such, excluded from the study as it was not possible to distinguish the correctly registered b/tsDMARD. These errors are typically caused by record overlap when a new b/tsDMARD is added before a previous one is removed. These patients with registration errors were omitted from the study and accounted for 0.5% of the annual average of current users receiving b/tsDMARDs during the study period. See Supplementary Table 1 for further details.

Among non-naïve users, a few of the previous b/tsDMARD administration was attributed to either anakinra, secukinumab, or ustekinumab, but none of these non-naïve users were treated only with one of these drugs. Hence, their previous use did not impact the amount for naïve and non-naïve users.

### Tender ranking, medication analysis

Each year the NHPT provides a report of the outcome of the Norwegian pharmaceutical tender, including a ranked list of b/tsDMARDs where rank 1 (highest rank) represents the least expensive drug offered. While the rank list is publicly available, the drug cost is confidential and only available for those with a need-to-know, e.g., prescribing rheumatologists, health economists, or certain health administrators. This study presents these recommendations with permission, albeit without details on the specific drug cost. The terms cost, price, and expenditure are used interchangeably in this paper with no distinction in context.

### Statistical analysis

Continuous variables are presented as mean with range and categorical variables as percentages with range. The variables for the ten-year period were calculated with an average of the mean from individual years. Change and association between variables over the ten-year period were analyzed with a one-way analysis of variance (ANOVA) for continuous variables and the chi-square test for categorical variables (independent for the b/tsDMARD overall, TNFi, non-TNFi, and tsDMARD groups). Although Table 1 displays each group’s average size (N), the annual N used for preliminary calculations was derived from the current dataset. Details on the annual N can be found in Table 4. The proportion computations (in percentage) in Tables 2, 3 and 4 are derived from the total annual count (N) for each user group. Detailed explanations of the calculations are provided in the footnotes. No imputations were used for missing data. A p-value of < 0.05 was considered statistically significant. Statistical analyses were performed using Statistical Package for Social Sciences (SPSS) version 28.0 and Microsoft Excel. The graphical elements were developed using Microsoft Excel and supplemented using Adobe Photoshop.

**Table 1:** Demographic and disease characteristics in Norwegian rheumatoid arthritis patients currently using b/tsDMARDs during 2010–2019

	b/tsDMARDs 2010–2019 Average *N* = 3322			TNFi 2010–2019 Average *N* = 2292			Non-TNFi 2010–2019 Average *N* = 961			tsDMARDs 2017–2019 Average *N* = 234		
	Average of Mean, Range of Mean	Missing Data *Mean, Range*	*P*	Average of Mean, Range of Mean	Missing Data *Mean, Range*	*P*	Average of Mean, Range of Mean	Missing Data *Mean, Range*	*P*	Average of Mean, Range of Mean	Missing Data *Mean, Range*	*P*
**Demographics**												
Age (years)	59, 59–60	1%, 0–11%	0.035	58, 58–59	1%, 0–12%	0.064	62, 61–63	1%, 0–8%	0.057	58, 57–60	0%, 0–0%	0.129
Female	73%, 72–73%	1%, 0–11%	0.946	71%, 71–72%	1%, 0–12%	0.994	76%, 74–78%	1%, 0–8%	0.753	75%, 72–79%	0%, 0–0%	0.138
BMI (kg/m2)	26, 26–26	10%, 2–57%	< 0.001	26, 25–26	11%, 2–58%	< 0.001	26, 26–26	8%, 1–51%	0.616	27, 26–27	1%, 0–2%	0.218
Current Smokers	18%, 14–22%	8%, 1–49%	< 0.001	18%, 13–22%	9%, 1–51%	< 0.001	18%, 13–22%	7%, 1–44%	< 0.001	12%, 10–15%	1%, 0–2%	0.553
Disease Duration (years)	14, 13–15	0%, 0–0%	< 0.001	13, 12–14	0%, 0–0%	< 0.001	16, 15–17	0%, 0–0%	< 0.001	15, 13–17	0%, 0–0%	0.183
**Biomarkers**												
aCCP Positive	81%, 80–82%	27%, 14–42%	0.921	79%, 77–81%	27%, 15–42%	0.283	87%, 84–88%	27%, 15–42%	0.875	78%, 73–83%	12%, 8–16%	0.184
RF Positive	73%, 72–75%	45%, 28–61%	0.745	71%, 69–74%	44%, 29–60%	0.558	79%, 76–83%	47%, 30–66%	0.232	72%, 67–78%	22%, 20–24%	0.106
**Disease Activity**												
ESR (mm/h)	15, 13–19	26%, 21–32%	< 0.001	16, 14–18	26%, 21–32%	< 0.001	14, 11–19	27%, 21–32%	< 0.001	22, 19–25	32%, 29–37%	0.316
CRP (mg/L)	6.5, 5.7–8.4	19%, 16–27%	< 0.001	5.9, 5.1–7.5	19%, 15–27%	< 0.001	7.8, 6.2–11	19%, 13–26%	< 0.001	11, 8–15	19%, 16–22%	0.029
TJC28 (0–28)	2.4, 1.7–3.4	15%, 9–19%	< 0.001	2.0, 1.5–2.9	12%, 7–18%	< 0.001	3.3, 1.8–5.2	20%, 13–25%	< 0.001	4.0, 2.3–6.1	13%, 10–16%	< 0.001
SJC28 (0–28)	1.4, 0.8–2.3	15%, 9–19%	< 0.001	1.2, 0.7–1.9	12%, 7–18%	< 0.001	1.9, 0.8–3.9	20%, 13–25%	< 0.001	2.3, 1.2–3.4	13%, 10–16%	< 0.001
IGA (VAS 0-100 mm)	15, 12–18	40%, 35–50%	< 0.001	14, 11–16	40%, 34–51%	< 0.001	18, 12–25	41%, 34–49%	< 0.001	24, 15–35	28%, 23–36%	< 0.001
DAS28	2.7, 2.4–3.1	30%, 26–34%	< 0.001	2.5, 2.3–2.9	28%, 22–34%	< 0.001	3.0, 2.5–3.7	36%, 32–42%	< 0.001	3.3, 2.7–3.9	31%, 26–38%	< 0.001
**Patient-Reported Outcome Measures**												
PGA (VAS 0-100 mm)	32, 32–33	10%, 8–12%	0.333	30, 30–31	8%, 6–10%	0.652	38, 34–42	15%, 12–16%	< 0.001	43, 36–50	11%, 8–14%	0.003
Pain (VAS 0-100 mm)	32, 32–33	20%, 14–53%	0.329	30, 29–31	19%, 12–55%	0.351	37, 34–41	24%, 17–47%	< 0.001	41, 36–49	15%, 12–20%	0.025
Fatigue (VAS 0-100 mm)	38, 37–40	37%, 15–54%	0.002	36, 35–38	36%, 13–53%	0.041	44, 42–46	41%, 20–57%	0.604	48, 47–49	55%, 49–61%	0.921

Categorical variables are presented as percentages and continuous variables as mean. The presented data are average values from the displayed year within the shown drug category. Missing data are presented as mean with range. χ^2^ test for categorical variables and one-way ANOVA for continuous variables was used to test for differences during follow-up of ten years

*Abbreviations*: *b/tsDMARDs *Biologic and target synthetic disease-modifying antirheumatic drugs, *TNFi *Tumor Necrosis Factor Inhibitor, *tsDMARDs *Target synthetic DMARDs, *BMI *Body Mass Index, *aCCP  *Anti-cyclic citrullinated peptide, *RF *Rheumatoid Factor, *ESR *Erythrocyte Sedimentation Rate, *CRP *C-Reactive Protein, *TJC28 *Tender 28-Joint Count, *SJC28 *Swollen 28-Joint Count, *IGA *Investigators Global Assessment, *VAS *Visual Analog Scale (Measured 0-100), *DAS28 *Disease Activity Score, *PGA *Patient Global assessment

**Table 2:** Overview of annual naïve b/tsDMARD prescriptions shown in numbers and percentages

	2010	2011	2012	2013	2014	2015	2016	2017	2018	2019
Total (N)	378	424	421	385	356	368	400	418	408	409
TNFi	326 (86.2)	382 (90.1)	384 (91.2)	349 (90.6)	310 (87.1)	309 (84.0)	362 (90.5)	372 (89.0)	327 (80.1)	355 (86.8)
Infliximab R^a^	**63 (16.7)**	20 (4.7)	**14 (3.3)**	14 (3.6)	7 (2.0)	11 (3.0)	0 (0)	0 (0)	2 (0.5)	0 (0)
Infliximab^**CT−P13**^	NA	NA	NA	NA	**45 (12.6)**	**140 (38.0)**	**145 (36.3)**	**106 (25.4)**	**156 (38.2)**	24 (5.9)
Etanercept R^a^	47 (12.4)	295 (69.6)	273 (64.8)	62 (16.1)	26 (7.3)	18 (4.9)	25 (6.3)	5 (1.2)	5 (1.2)	0 (0)
Etanercept^**SB4**^	NA	NA	NA	NA	NA	NA	22 (5.5)	208 (49.8)	138 (33.8)	13 (3.2)
Adalimumab R^a^	63 (16.7)	18 (4.2)	10 (2.4)	5 (1.3)	7 (2)	1 (0.3)	3 (0.8)	1 (0.2)	5 (1.2)	**313 (76.5)**
Certolizumab	12 (3.2)	21 (5.0)	69 (16.4)	**249 (64.7)**	215 (60.4)	134 (36.4)	166 (41.5)	52 (12.4)	19 (4.7)	5 (1.2)
Golimumab	141 (37.3)	28 (6.6)	18 (4.3)	19 (4.9)	10 (2.8)	5 (1.4)	1 (0.3)	0 (0)	2 (0.5)	0 (0)
Non-TNFi	52 (13.8)	42 (9.9)	37 (8.8)	36 (9.4)	46 (12.9)	59 (16.0)	38 (9.5)	46 (11.0)	51 (12.5)	23 (5.6)
Abatacept^a^	8 (2.1) §	4 (0.9)	0 (0)	3 (0.8)	2 (0.6)	8 (2.2)	4 (1.0)	9 (2.2)	9 (2.2)	1 (0.2)
Rituximab R^a^	29 (7.7)^b^	**34 (8.0)**^b^	19 (4.5)^b^	16 (4.2)^b^	21 (5.9)^b^	19 (5.2)^b^	22 (5.5)	18 (4.3)	26 (6.4)^b^	7 (1.7)^b^
Rituximab^**GP2013**^	NA	NA	NA	NA	NA	NA	NA	NA	0 (0)	12 (2.9)^b^
Tocilizumab^a^	15 (4.0)	4 (0.9)	18 (4.3)	17 (4.4)	23 (6.5)	32 (8.7)	12 (3.0)	19 (4.5)	16 (3.9)	3 (0.7)
tsDMARDs	NA	NA	NA	NA	NA	NA	NA	0 (0)	30 (7.4)	31 (7.6)
Tofacitinib	NA	NA	NA	NA	NA	NA	NA	0 (0)	30 (7.4)	3 (0.7)
Baricitinib	NA	NA	NA	NA	NA	NA	NA	0 (0)	0 (0)	28 (6.8)
∑ 1st + 2nd HV	54%^c^	78%^c^	81%	81%^c^	73%^c^	74%^c^	78%^c^	75%^c^	72%^c^	83%^c^
∑ SC + PO	70%	85%	88%	87%	73%	43%	54%	64%	49%	89%
∑ Biosimilar	0%	0%	0%	0%	13%	38%	42%	75%	72%	12%

Annual naïve treatment of rheumatoid arthritis patients in Norway for the period 2010–2019 showing the prescription of specific b/tsDMARDs. Total (N) is the amount of naïve b/tsDMARD prescriptions each year. All values are the annual numbers of prescribed drugs (or subcategories) with a percentage (%) of the Total (N). ∑ 1st + 2nd HV = The sum of the first and second Highest Proportion of b/tsDMARDs. ∑ SC + PO = The accumulated amount of subcutaneous and per oral b/tsDMARD of all naïve b/tsDMARD users. ∑ Biosimilar = The sum of the total amount of biosimilars

*Abbreviation*: *b/tsDMARDs *Biologic and targeted synthetic disease-modifying antirheumatic drugs, *TNFi *Tumor Necrosis Factor inhibitor, *tsDMARDs *Target synthetic DMARDs, *NA *Not available, *R *Reference agent

^a^Infliximab, etanercept, adalimumab reference, abatacept, and rituximab had their first recommendation in 2008 and tocilizumab in 2009

^b^Illustrates which drug was recommended on the condition of being a second-line drug

^c^Indicate the tender winner is also either the highest or second highest in proportion. The annual tender winner is marked in bold

**Table 3:** Overview of annual non-naïve b/tsDMARDs prescription shown in numbers and percentages

	2010	2011	2012	2013	2014	2015	2016	2017	2018	2019
Total (N)	452	463	454	471	496	578	1270	921	656	1065
TNFi	213 (47.1)	252 (54.4)	282 (62.1)	281 (59.7)	278 (56.0)	363 (62.8)	1052 (82.8)	635 (68.9)	333 (50.8)	517 (48.5)
Infliximab R^b^	**23 (5.1)**	11 (2.4)	**24 (5.3)**	17 (3.6)	12 (2.4)	10 (1.7)	0 (0)	0 (0)	1 (0.2)	0 (0)
Infliximab^CT−P13^	NA	NA	NA	NA	**29 (5.8)**	**159 (27.5)**	**355 (28.0)**	**213 (23.1)**	**173 (26.4)**	122 (11.5)
Etanercept R^b^	60 (13.3)	98 (21.2)	62 (13.7)	44 (9.3)	43 (8.7)	37 (6.4)	37 (2.9)	4 (0.4)	2 (0.3)	1 (0.1)
Etanercept^**SB4**^	NA	NA	NA	NA	NA	NA	494 (38.9)	360 (39.1)	127 (19.4)	57 (5.4)
Adalimumab R^b^	36 (8.0)	41 (8.9)	22 (4.8)	10 (2.1)	17 (3.4)	6 (1)	5 (0.4)	3 (0.3)	8 (1.2)	**320 (30.0)**
Certolizumab	32 (7.1)	77 (16.6)	138 (30.4)	**152 (32.3)**	137 (27.6)	118 (20.4)	144 (11.3)	42 (4.6)	17 (2.6)	14 (1.3)
Golimumab	62 (13.7)	25 (5.4)	36 (7.9)	58 (12.3)	40 (8.1)	33 (5.7)	17 (1.3)	13 (1.4)	5 (0.8)	3 (0.3)
Non-TNFi	239 (52.9)	211 (45.6)	172 (37.9)	190 (40.3)	218 (44.0)	215 (37.2)	218 (17.2)	231 (25.1)	133 (20.3)	321 (30.1)
Abatacept^b^	43 (9.5)^c^	38 (8.2)	15 (3.3)	10 (2.1)	51 (10.3)	59 (10.2)	47 (3.7)	57 (6.2)	32 (4.9)	11 (1.0)
Rituximab R^b^	125 (27.7)^c^	**117 (25.3)**^c^	83 (18.3)^c^	94 (20.0)^c^	101 (20.4)^c^	87 (15.1)^c^	91 (7.2)	90 (9.8)	65 (9.9)^c^	10 (0.9)^c^
Rituximab^**GP2013**^	NA	NA	NA	NA	NA	NA	NA	NA	3 (0.5)	284 (26.7)^c^
Tocilizumab^b^	71 (15.7)	56 (12.1)	74 (16.3)	86 (18.3)	66 (13.3)	69 (11.9)	80 (6.3)	84 (9.1)	33 (5.0)	16 (1.5)
tsDMARDs	NA	NA	NA	NA	NA	NA	NA	55 (6.0)	190 (29.0)	227 (21.3)
Tofacitinib	NA	NA	NA	NA	NA	NA	NA	39 (4.2)	179 (27.3)	29 (2.7)
Baricitinib	NA	NA	NA	NA	NA	NA	NA	16 (1.7)	11 (1.7)	198 (18.6)
∑ 1st + 2nd HV	43%	47%^a^	49%	52%^a^	48%	48%^a^	67%^a^	62%^a^	54%^a^	57%^a^
∑ SC + PO	42%	52%	57%	56%	48%	34%	55%	52%	53%	58%
∑ Biosimilar	NA	NA	NA	NA	6%	28%	67%	62%	46%	44%

Annual non-naïve treatment of rheumatoid arthritis patients in Norway for the period 2010–2019 showing the prescription of specific b/tsDMARDs. Total (N) is the amount of non-naïve b/tsDMARD prescriptions each year. All values are the annual numbers of prescribed drugs (or subcategories) with a percentage (%) of the Total (N). ∑ 1st + 2nd HV = The sum of the first and second Highest Proportion of b/tsDMARDs. ∑ SC + PO = The accumulated amount of subcutaneous and per oral b/tsDMARD of all naïve b/tsDMARD users. ∑ Biosimilar = The sum of the total amount of biosimilars

*Abbreviation*: *b/tsDMARDs *Biologic and targeted synthetic disease-modifying antirheumatic drugs, *TNFi *Tumor Necrosis Factor inhibitor, *tsDMARDs *Target synthetic DMARDs, *NA *Not available, *R *Reference agent

^a^Indicate the tender winner is also either the highest or second highest in proportion. The annual tender winner is marked in bold

^b^Infliximab, etanercept, adalimumab reference, abatacept, and rituximab had their first recommendation in 2008 and tocilizumab in 2009

^c^Illustrates which drug was recommended on the condition of being a second-line drug

**Table 4:** Overview of annual current b/tsDMARD use shown in amount and percentage

	2010	2011	2012	2013	2014	2015	2016	2017	2018	2019
	*n* = 4885	*n* = 7230	*n* = 7970	*n* = 7248	*n* = 7993	*n* = 9010	*n* = 9037	*n* = 9129	*n* = 9048	*n* = 9280
Total (N)	1910 [39.1]	2829 [39.1]	3111 [39.0]	3029 [41.8]	3388 [42.4]	3639 [40.4]	3631 [40.2]	3771 [41.3]	3813 [42.1]	4098 [44.2]
TNFi	1454 (76.1)	2099 (74.2)	2279 (73.3)	2212 (73.0)	2430 (71.7)	2470 (67.9)	2442 (67.3)	2498 (66.2)	2394 (62.8)	2639 (64.4)
Infliximab R^b^	**234 (12.3)**	274 (9.7)	**252 (8.1)**	242 (8.0)	238 (7.0)	152 (4.2)	63 (1.7)	38 (1.0)	27 (0.7)	18 (0.4)
Infliximab^CT−P13^	NA	NA	NA	NA	**60 (1.8)**	**269 (7.4)**	**435 (12.0)**	**448 (11.9)**	**510 (13.4)**	438 (10.7)
Etanercept R^b^	688 (36.0)	1130 (39.9)	1242 (39.9)	1104 (36.4)	1078 (31.8)	977 (26.8)	620 (17.1)	226 (6.0)	164 (4.3)	113 (2.8)
Etanercept^SB4^	NA	NA	NA	NA	NA	NA	255 (7.0)	874 (23.2)	948 (24.9)	878 (21.4)
Adalimumab R^b^	396 (20.7)	430 (15.2)	428 (13.8)	315 (10.4)	313 (9.2)	295 (8.1)	262 (7.2)	225 (6.0)	200 (5.2)	**713 (17.4)**
Certolizumab	29 (1.5)	94 (3.3)	188 (6.0)	**374 (12.3)**	553 (16.3)	611 (16.8)	660 (18.2)	571 (15.1)	444 (11.6)	394 (9.6)
Golimumab	107 (5.6)	171 (6.0)	169 (5.4)	177 (5.8)	188 (5.5)	166 (4.6)	147 (4.0)	116 (3.1)	101 (2.6)	85 (2.1)
Non-TNFi	456 (23.9)	730 (25.8)	832 (26.7)	817 (27.0)	958 (28.3)	1169 (32.1)	1189 (32.7)	1242 (32.9)	1169 (30.7)	1047 (25.5)
Abatacept^b^	68 (3.6) §	85 (3.0)	79 (2.5)	68 (2.2)	97 (2.9)	126 (3.5)	129 (3.6)	147 (3.9)	125 (3.3)	99 (2.4)
Rituximab R^b^	329 (17.2)^c^	**533 (18.8)**^c^	589 (18.9)^c^	558 (18.4)^c^	617 (18.2)^c^	753 (20.7)^c^	777 (21.4)	788 (20.9)	781 (20.5)^c^	456 (11.1)^c^
Rituximab^GP2013^	NA	NA	NA	NA	NA	NA	NA	NA	4 (0.1)	266 (6.5)^c^
Tocilizumab^b^	59 (3.1)	112 (4.0)	164 (5.3)	191 (6.3)	244 (7.2)	290 (8.0)	283 (7.8)	307 (8.1)	259 (6.8)	226 (5.5)
tsDMARDs	NA	NA	NA	NA	NA	NA	NA	31 (0.8)	250 (6.6)	412 (10.1)
Tofacitinib	NA	NA	NA	NA	NA	NA	NA	24 (0.6)	233 (6.1)	227 (5.5)
Baricitinib	NA	NA	NA	NA	NA	NA	NA	7 (0.2)	17 (0.4)	185 (4.5)
∑ 1st + 2nd HV	57%	59%^a^	59%	55%	50%	48%	40%	44%	45%	39%^a^
∑ SC + PO	64%	65%	65%	65%	63%	56%	54%	54%	55%	63%
∑ Biosimilar	NA	NA	NA	NA	2%	7%	19%	35%	38%	39%

Annual current treatment of rheumatoid arthritis patients in Norway for the period 2010–2019 showing the use of specific b/tsDMARDs. Total (N) is the amount of current b/tsDMARD use each year. All values are the annual numbers of used drugs (or subcategories) with a percentage (%) of the Total (N). Percentages in brackets [%] are estimated from the total registered patients (n). ∑ 1st + 2nd HV = The sum of the first and second Highest Proportion of b/tsDMARDs. ∑ SC + PO = The accumulated amount of subcutaneous and per oral b/tsDMARD of all naïve b/tsDMARD users. ∑ Biosimilar = The sum of the total amount of biosimilars

*Abbreviation: b/tsDMARDs *Biologic and targeted synthetic disease-modifying antirheumatic drugs, *TNFi *Tumor Necrosis Factor inhibitor, *tsDMARDs *Target synthetic DMARDs, *NA *Not available, *R *Reference agent

^a^Indicate the tender winner is also either the highest or second highest in proportion. The annual tender winner is marked in bold

^b^Infliximab, etanercept, adalimumab reference, abatacept, and rituximab had their first recommendation in 2008 and tocilizumab in 2009

^c^Illustrates which drug was recommended on the condition of being a second-line drug

### Ethics

The study was approved by the regional ethical committee for medical health research ethics (REC) (Regional etisk komite Midt-Norge 2010/3078) and consequently follows the Declaration of Helsinki ethical principles of medical research involving human subjects. The study was also approved by the Institutional Review Board (Research Unit Sørlandet Hospital) and met the requirements of the Health Research Act [Helseforksningsloven] from 2009. The protocol used anonymized data, which did not require confirmed consent from the patient and was approved by the regional ethical committee for medical health research ethics. All data was collected as part of routine clinical care.

## Results

### Demographics and disease characteristics

Table 1 displays aggregated data on demographics, disease activity, and patient-reported outcomes based on treatment groups to contextualize the b/tsDMARD data assessments better and understand the evaluated RA population. The aggregated average values among current b/tsDMARD users were 59 years of age, 73% females, 26 kg/m^2^ in BMI, 18% smokers, 14 years disease duration, 2.7 in DAS28, and 32 VAS in PGA. Table 1 shows further details of the aggregated overall b/tsDMARDs users and the three subgroups TNFi, non-TNFi, and tsDMARDs. Annual data on current, naïve, and non-naïve b/tsDMARD users is presented elsewhere [16].

### The proportion of b/tsDMARDs

**Fig. 2:**
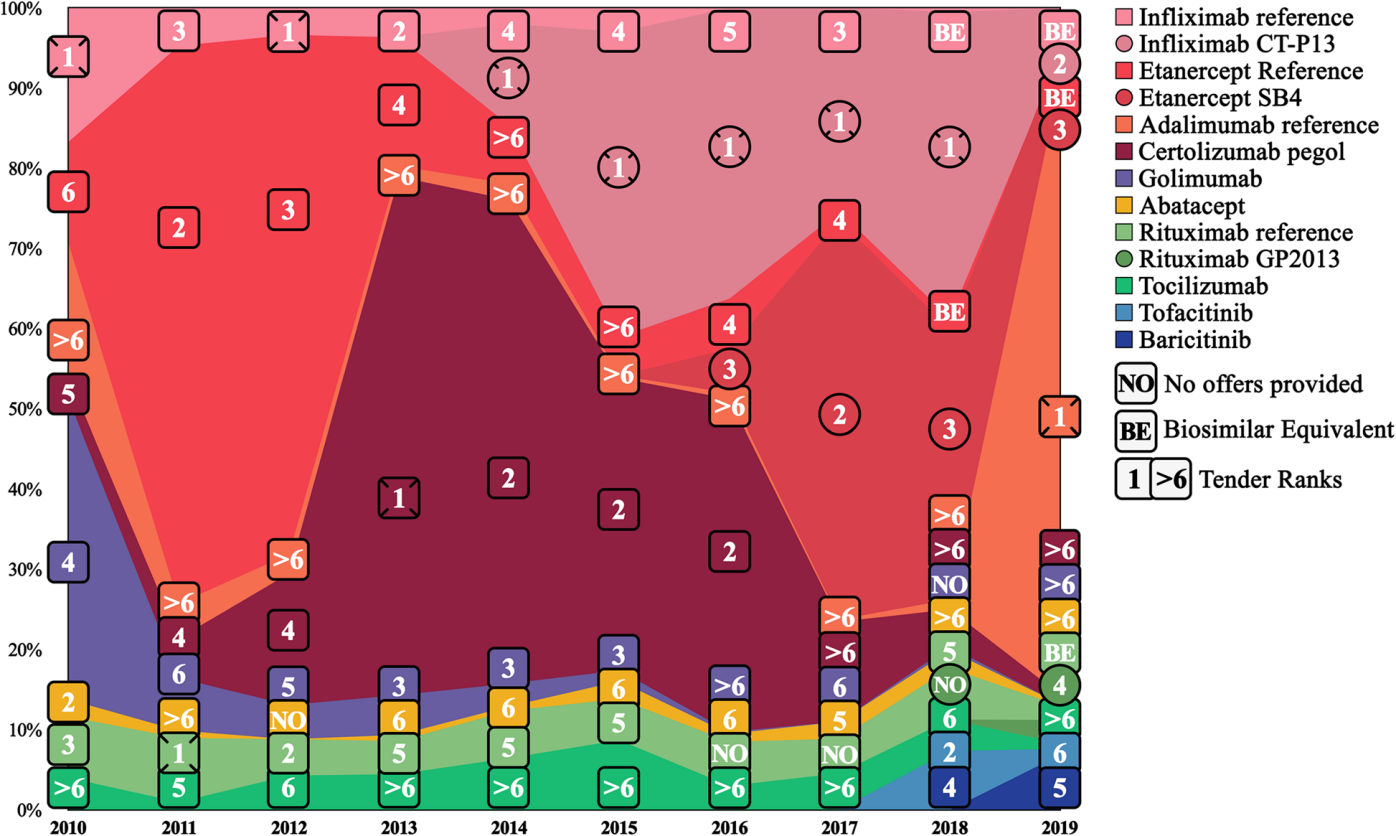
Overview of annual naïve b/tsDMARD prescriptions illustrated with tender rankings in a stacked area graph. *Note*: Annual naïve treatment of rheumatoid arthritis patients in Norway for the period 2010–2019 showing the prescription of specific b/tsDMARDs drugs with corresponding tender rankings for the b/tsDMARDs drugs displayed using a stacked area graph. The specific values are shown in Table 3. Each b/tsDMARD has a unique color, shown on the right. Biosimilars are marked with circles. No offers indicate b/tsDMARD refrained from participating in the annual tender. Only the best offer was recommended among participating equivalent biosimilars and their corresponding reference agents. Those that provided an offer but were not recommended are marked as Biosimilar Equivalents. *Abbreviation*: b/tsDMARDs = biologic and targeted synthetic disease-modifying antirheumatic drugs

Table 2 shows an overview of b/tsDMARD prescriptions among naïve users, while Fig. 2 displays the annual tender ranking and percentage visualization of naïve prescriptions. Over the ten years, the total number of annual b/tsDMARD prescriptions for naïve users increased from 378 to 409 (highest in 2017 with 418). A proportion change from 86.2% to 2010 to 86.8% in 2019 (highest in 2012 with 91.2%) for TNFi and a proportion decrease from 13.8% to 2010 to 5.6% in 2019 (highest in 2015 with 16.0%) for non-TNFi was observed. An increase of 7.6% for tsDMARDs was observed in the last three years. No tsDMARD use was registered prior to 2017.

**Fig. 3:**
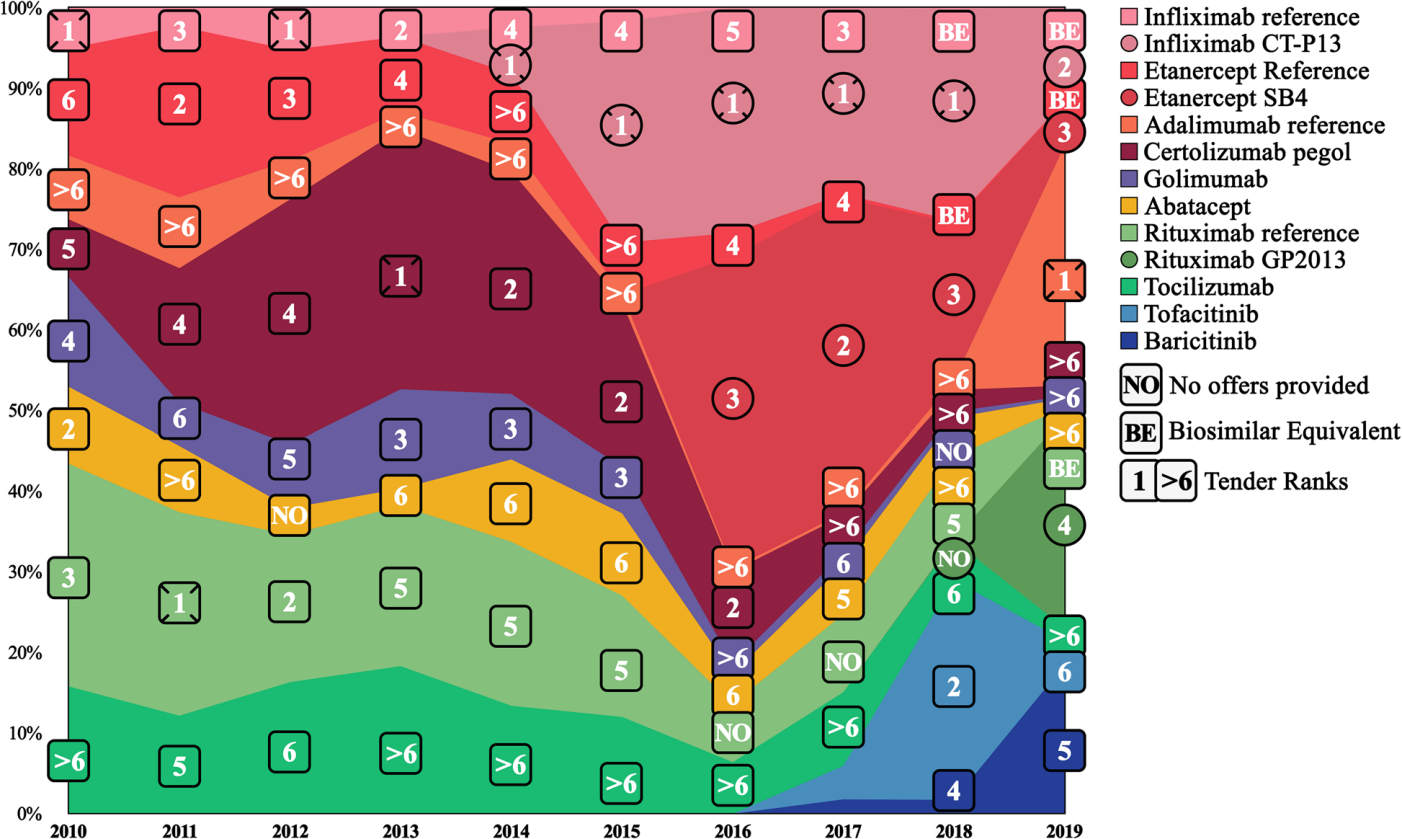
Overview of annual non-naïve b/tsDMARD prescriptions illustrated with tender rankings in a stacked area graph. *Note*: Annual non-naïve treatment of rheumatoid arthritis patients in Norway for the period 2010–2019 showing the prescription of specific b/tsDMARDs drugs with corresponding tender rankings for the b/tsDMARDs drugs displayed using a stacked area graph. The specific values are shown in Table 4. Each b/tsDMARD has a unique color, shown on the right. Biosimilars are marked with circles. No offers indicate b/tsDMARD refrained from participating in the annual tender. Only the best offer was recommended among participating equivalent biosimilars and their corresponding reference agents. Those that provided an offer but were not recommended are marked as Biosimilar Equivalents. *Abbreviation*: b/tsDMARDs = biologic and targeted synthetic disease-modifying antirheumatic drugs

Table 3 shows an overview of b/tsDMARD proportion among non-naïve users, while Fig. 3 displays the annual tender ranking and percentage visualization for the non-naïve users. Over the ten years, the total annual b/tsDMARD proportion for non-naïve users increased from 452 to 1065. A proportion change from 47.1% to 2010 to 48.5% in 2019 (highest in 2016 with 82.8%) for TNFi and a proportion decrease from 52.9% to 2010 to 30.1% in 2019 (highest in 2010) for non-TNFi was observed. The tsDMARDs increased to 21.3% in 2019 (29.0% in 2018).

**Fig. 4:**
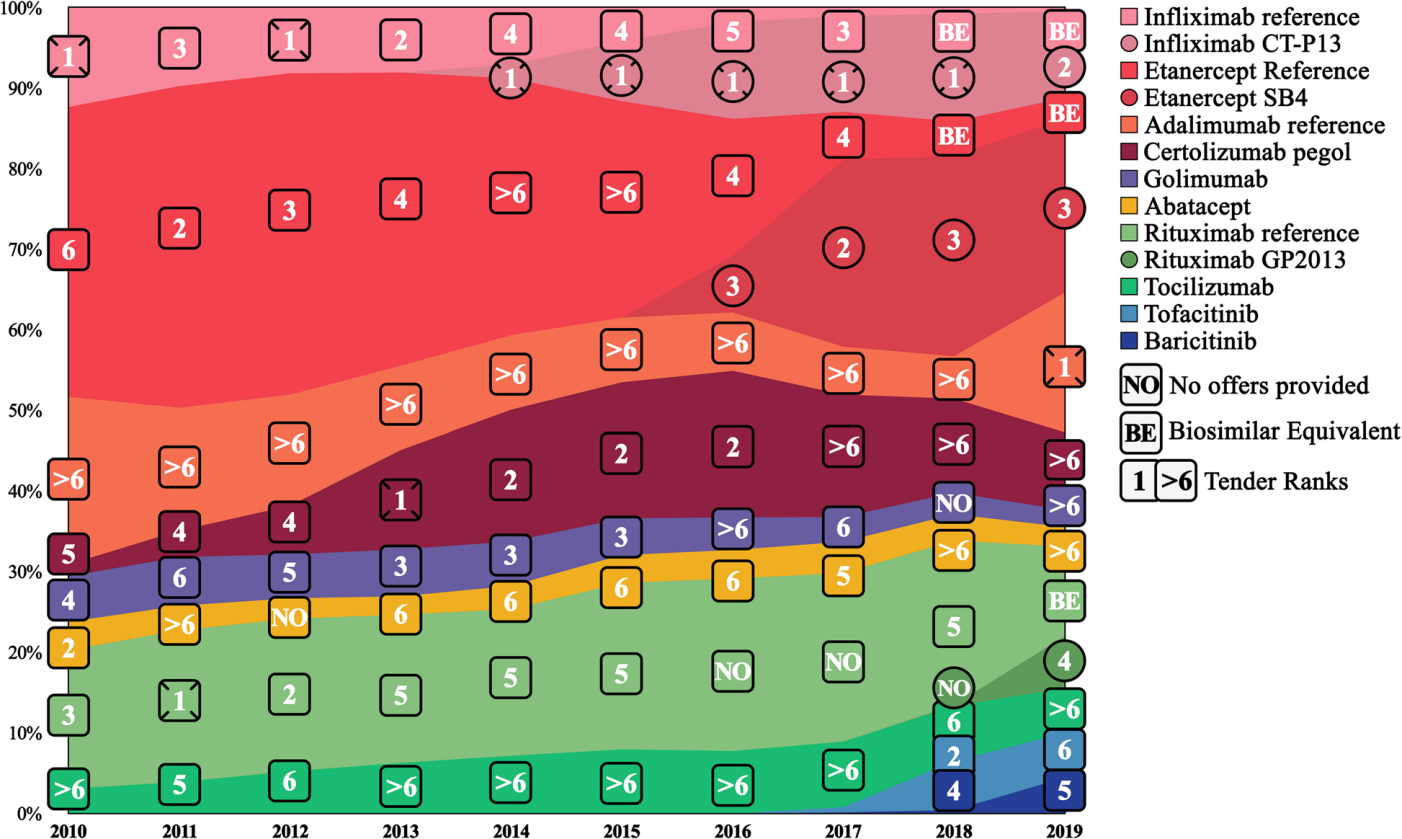
Overview of annual current b/tsDMARDs use illustrated with tender rankings in a stacked area graph. *Note*: Annual current treatment of rheumatoid arthritis patients in Norway for the period 2010–2019 showing the use of specific b/tsDMARDs drugs with corresponding tender rankings for the b/tsDMARDs drugs displayed using a stacked area graph. The specific values are shown in Table 2. Each b/tsDMARD has a unique color, shown on the right. Biosimilars are marked with circles. No offers indicate b/tsDMARD refrained from participating in the annual tender. Only the best offer was recommended among participating equivalent biosimilars and their corresponding reference agents. Those that provided an offer but were not recommended are marked as Biosimilar Equivalents. *Abbreviation*: b/tsDMARDs = biologic and targeted synthetic disease-modifying antirheumatic drugs

During the ten-year study period, the number of registered RA patients in the databases increased from 4885 to 2010 to 9280 in 2019. Table 4 reports the number of current users of b/tsDMARDs, while Fig. 4 shows the annual tender ranking and visualizes the current b/tsDMARD proportion. The percentage of annual individual b/tsDMARD use increased from 39.1% (*n* = 1910) in 2010 to 44.2% (*n* = 4098) in 2019. Across the study period, the TNFi proportion decreased from 76.1% to 2010 to 64.4% in 2019 (highest in 2010), non-TNFi increased from 23.9% to 2010 to 25.5% in 2019 (highest in 2017 with 32.9%), while tsDMARDs increased to 10.1% in 2019.

The average sum of the highest and second highest proportions among naïve, non-naïve, and current users were 75%, 53%, and 50% during the ten years, respectively. In Figs. 2, 3 and 4, the ranks are arranged from 1 to 6 ((1), (2), (3), (4), (5), and (6)), ranks > 6 (> 6), those that did not give any offers (NO), and those that were outcompeted by their equivalent biosimilar (BE). The annual winner for each year is emboldened in Tables 2, 3 and 4.

### Individual b/tsDMARD proportion observations

Rituximab reference (iv) was approved in 1998 and included in the Norwegian pharmaceutical tendering. Initially, rituximab reference remained relatively stable across all user categories, then decreased over six years among naive and non-naive but not current users. Despite this pattern, rituximab reference was either the highest or second highest in proportion in eight out of ten years for current users, five for non-naïve users, and once for naïve users.

During the first four years (2010–2013), iv infliximab reference (approved in 1999) was observed to have a low prescription proportion compared with all other b/tsDMARDs. In 2014 its biosimilar infliximab CT-P13 gave its first offer (ranking first). In the following years (2014 to 2016), infliximab reference fell close to zero in all categories, while infliximab CT-P13 rapidly increased. Infliximab CT-P13 decreased drastically in 2019 for both naïve and non-naïve users. That same year iv biosimilar rituximab GP2013 was introduced, and sc adalimumab reference won the tendering. While rituximab GP2013 was negligible for the naïve and current users, it acquired a quarter of the non-naïve proportion in 2019 (rank 4).

Etanercept reference (sc) was also one of the b/tsDMARDs that participated in the early years of pharmaceutical tendering, and in contrast to most other b/tsDMARD, etanercept reference managed to acquire a large proportion prior to 2010. In 2010, it constituted 36% of the total current proportion. This pattern remained stable at around one-third of the total and as the highest proportion until the introduction of etanercept SB4 in 2016, after which etanercept reference gradually decreased (3% in 2019) while etanercept SB4 increased. While not equally stable but overall high in proportion, the same turnover between etanercept reference and its biosimilar was observed among naïve and non-naïve users.

The second observably high sc b/tsDMARD was certolizumab pegol. While it did not reach high numbers initially nor ever managed to reach the highest proportion among current users (2–18%), its proportion was relatively stable, even after the introduction of biosimilars. It was most prescribed between 2012 and 2016 for naïve and non-naïve users, acquiring the highest or second highest proportion in almost all those years. Between 2013 and 2016, it provided either the best or second-best offer during the tendering. Interestingly, from 2017 to 2019, its offers were above rank six each year, but it still managed to keep relatively stable current users.

Golimumab (sc) never acquired a large proportion and never came first or second on the tender ranking (three times rank 3). Adalimumab reference (sc), which had even worse-ranked offers (all above rank six, except 2019), had a higher proportion among current users but lower among naïve and non-naïve compared to golimumab. In 2019 adalimumab reference was priced lower than its corresponding biosimilar adalimumab GP2017, Hyrimoz®, and ranked first in the pharmaceutical tendering (resulting in an exclusion of Hyrimoz from the Norwegian tendering). During the same year, a slight increase (5–17%) in proportion was observed among the adalimumab reference current users, and a substantial increase was observed among both the naïve (1–77% increase in proportion) and non-naïve users (1–30% increase).

Tocilizumab (iv) proportion increased during the first four years but gradually decreased during the next six years for naïve and non-naïve users. For current users, it remained stable after the initial increase. Between 2010 and 2019, abatacept (iv) had a low yet stable prescription proportion for all categories, with offers in the tendering ranking above fifth for most years. Both tocilizumab and abatacept were initially offered as iv drugs, but from 2014 (tocilizumab) and 2012 (abatacept), they were also approved for subcutaneous use.

Rituximab reference was only recommended as a second-line drug during the ten years, except those years when no offers were provided. Abatacept in 2010 and rituximab GP2013 in 2019 were also recommended as second-line drugs. In other words, these drugs were recommended to be used only when another b/tsDMARD showed inadequate effect or resulted in adverse effects. Second-line drugs are marked in Tables 2, 3 and 4.

Tofacitinib and baricitinib were introduced in 2017 as po (tablet) b/tsDMARDs (tsDMARDs). Non-naïve users in 2018 for tofacitinib and 2019 for baricitinib acquired about 30% and 20% of the total proportion, respectively. The tsDMARDs did not acquire more than 10% combined proportion in the other user subgroups and years.

### Subcutaneous and oral vs. intravenous b/tsDMARD proportion

**Fig. 5:**
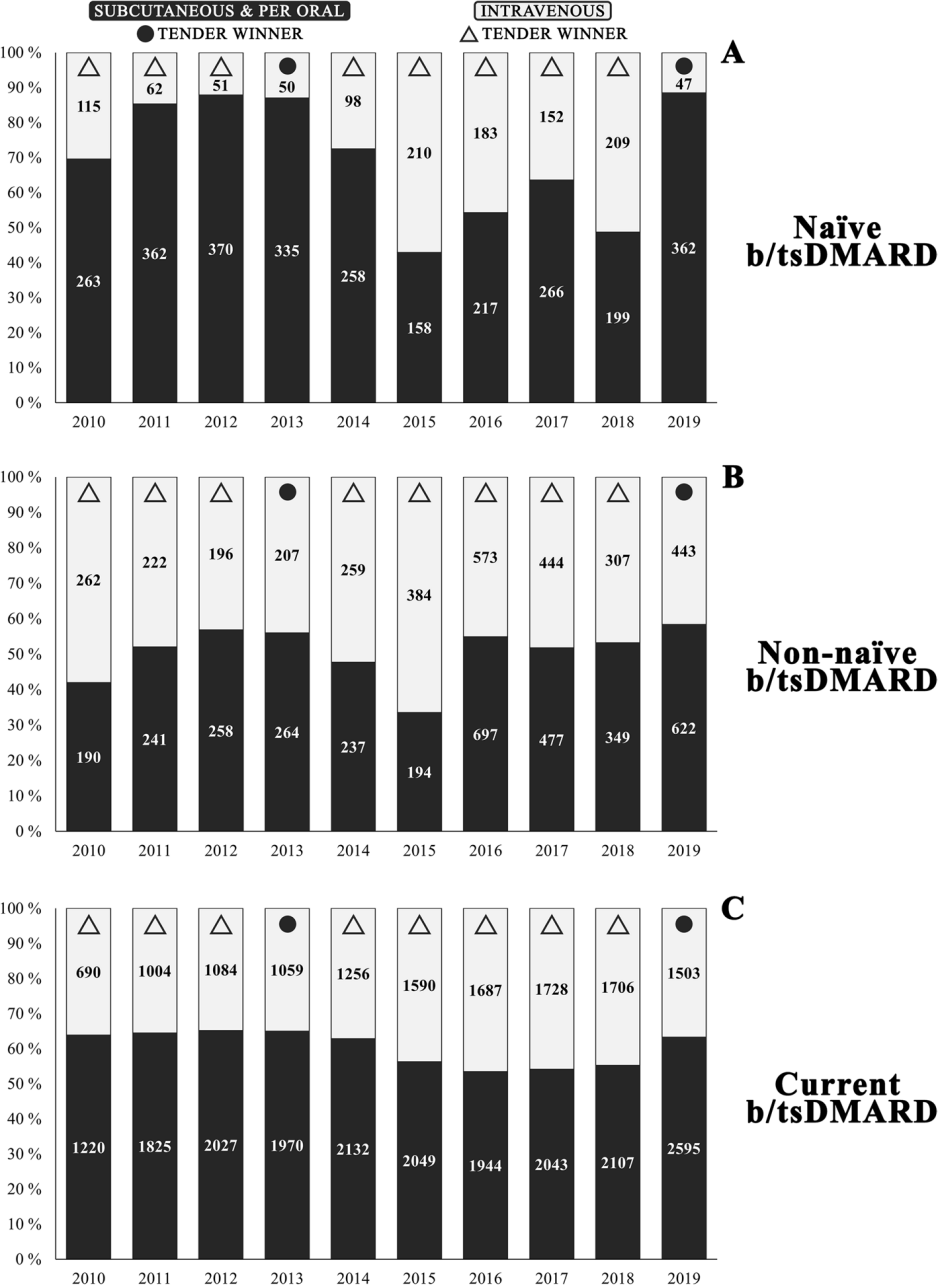
b/tsDMARD route of administration comparison for naïve (**A**), non-naïve (**B**), and current (**C**) treatment. *Note*: Overview comparison between intravenous and subcutaneous and tablet b/tsDMARDs for the subcategories naïve (**A**), non-naïve (**B**), and current (**C**) treatment of rheumatoid arthritis patients in Norway for the period 2010–2019. Circular icons illustrate if a subcutaneous or per oral b/tsDMARD was the tender-winning drug. Triangle icons illustrate if an intravenous b/tsDMARD was a tender-winning drug. The specific values are shown in Tables 2, 3, and 4. *Abbreviation*: b/tsDMARDs = biologic and targeted synthetic disease-modifying antirheumatic drugs

On average, across the ten years, the sum of subcutaneous and per oral (sc/po) b/tsDMARDs accounted for 70.0% (range 42.9–88.5%) among all naïve b/tsDMARD users, 50.7% (range 33.6–58.4%) among non-naïve users, and 60.4% (range 53.5–62.2%) among current users (Fig. 5).

Etanercept reference and its biosimilar SB4 combined and certolizumab pegol constituted about 40% on average each of the sc/po b/tsDMARD proportion among naïve users and around 35% on average among non-naïve users. The combined proportion of etanercept reference and its biosimilar were highest for the current users, where it constituted 52% on average of the sc/po b/tsDMARD proportion. While adalimumab reference achieved 11–18% on average among the different subgroup users, it constituted 86%, 51%, and 28% of the sc/po b/tsDMARD proportion for naïve, non-naïve, and current users in 2019, respectively.

The iv b/tsDMARDs accounted for an average of 30.0% (range 11.5–57.1%) among all naïve b/tsDMARD users, 49.3% (range 41.6–66.4%) among non-naïve users, and 39.6% (range 53.5–62.2%) among current users (Fig. 5). For naïve users, the combined proportion of infliximab reference and infliximab CT-P13 constituted 54% on average of all iv b/tsDMARDs. In comparison, the rituximab reference and rituximab GP2013 combined covered less than 2% on average. For non-naïve users, the infliximab combination and the rituximab combination were relatively similar on average (29% vs. 37%, respectively). Among non-naïve iv b/tsDMARD users, Rituximab reference dominated until infliximab CT-P13 started gaining market in 2014–2015, with another shift in 2019 in favor of rituximab GP2013. For current users, the combination difference was more prominent, with 49% for the rituximab combination and 28% for the infliximab combination.

While sc/po b/tsDMARD were consistently higher or similar in proportion compared to iv b/tsDMARDs across all three user subgroups, the tender-winning drug was iv in eight of ten pharmaceutical tenders. For iv current and non-naïve users, the rituximab combination was favored over the infliximab combination, despite the infliximab combination winning the tender seven out of ten times, while the rituximab combination won once. The accumulated sc/po b/tsDMARDs are shown separately in Tables 2, 3, and 4.

### Biosimilars vs. non-biosimilar proportion

**Fig. 6:**
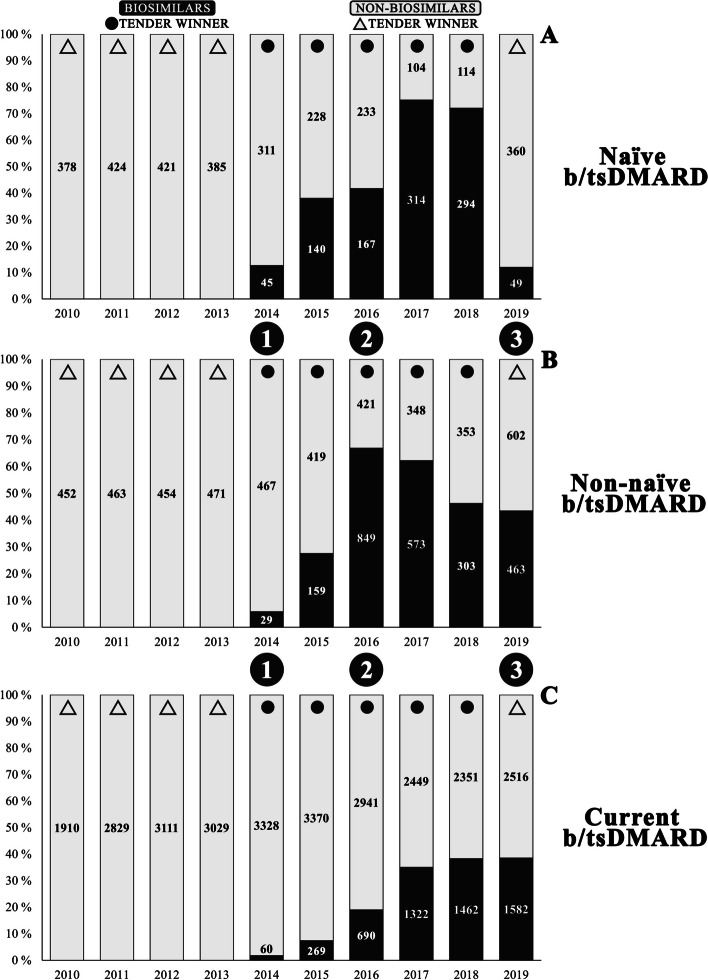
b/tsDMARD biosimilar vs. non-biosimilar comparison for naïve (**A**), non-naïve (**B**), and current (**C**) treatment. *Note*: Overview comparison between intravenous and subcutaneous and tablet b/tsDMARDs for the subcategories naïve (**A**), non-naïve (**B**), and current (**C**) treatment of rheumatoid arthritis patients in Norway for the period 2010–2019. Circular icons illustrate if a biosimilar was a tender-winning drug. Triangle icons illustrate if a non-biosimilar was a tender-winning drug. The large circles with numbers 1, 2, and 3 mark the introduction of the first, second, and third biosimilar, respectively. The specific values are shown in Tables 2 and 3, and 4. *Abbreviation*: b/tsDMARDs = biologic and targeted synthetic disease-modifying antirheumatic drugs

During the last six years of the study period, biosimilars were observed to have either the highest or second highest proportion six out of six years for naïve b/tsDMARD users, five for non-naïve b/tsDMARD users, and three for current b/tsDMARD users. The average total biosimilar proportion was 41.9% (range 12.0–75.1%), 42.0% (range 5.8–66.9%), and 23.4% (range 1.8–38.6%) for naïve, non-naïve and current b/tsDMARD users, respectively (Tables 2, 3 and 4). The tender-winning b/tsDMARD was biosimilar infliximab CT-P13 in five out of six tenders. Observations of the turnover between the reference agent (infliximab and etanercept) and their corresponding biosimilars in all user subgroups can be found in Figs. 2, 3 and 4. The difference between biosimilars and non-biosimilar is displayed in Fig. 6.

## Discussion

The main finding of this study shows that b/tsDMARD procurements during the ten-year period, guided by the Norwegian pharmaceutical tendering, appeared to influence the choice of treatment among RA patients registered on a new b/tsDMARD, especially among those without prior b/tsDMARD registration (naïve users). This was determined by observing a tender-winning b/tsDMARD also being the highest or second highest in registration in nine out of ten years for naïve users, seven out of ten for non-naïve users, and only twice among current users. While the iv b/tsDMARDs were the tender-winning drug in seven out of ten years, the sc/po b/tsDMARDs acquired a higher proportion on average among naïve and current users and equally among non-naïve users. Based on these observations, generalized assumptions can be made. A better offer (i.e., higher tender rank) appears to be linked with increased new registrations for the given b/tsDMARD. However, the highest tender rank may not always correspond with changes in b/tsDMARD registration pattern among the current b/tsDMARD users. This may be due to the high number of loyal b/tsDMARD users or prescribers, i.e., those patients who wish to remain or are kept by their rheumatologist on the same b/tsDMARD despite better economic alternatives.

One may argue that if the loyalty of either the user or the prescriber to the given b/tsDMARD is established, e.g., via consistent low cost and/or treatment satisfaction, it is more likely that the RA patient will remain on their current b/tsDMARD, regardless of less costly alternatives available. Such an example can be observed with certolizumab pegol, which gained the proportion benefit among naïve and non-naïve users with first- and second-rank offers in the period between 2013 and 2016. Thereafter, during the three following years, certolizumab pegol remained relatively stable in proportion among current users despite only providing tender offers above rank six. This loyalty is further endorsed by the lack of available evidence or recommendations to promote a cost-beneficial non-medical switch between various b/tsDMARDs with different substances (e.g., within TNFi, or between TNFi and non-TNFi) [18]. Norwegian rheumatologists generally follow the “do not change from the winning team” principle. However, switching non-medically between a reference agent and its biosimilar (or between biosimilars) is now generally recognized and is usually conducted to achieve lower costs in Norway. Some may argue that there is inconclusive data on the safety of conducting this type of non-medical switch and advocate for not switching between reference agent and biosimilar; however, no consistent evidence suggests it is unsafe either [19]. Results from Norway (NOR-SWITCH study) [20, 21] and Denmark (DANBIO registry) [22, 23] have shown positive outcomes when switching (even non-medically) patients from reference agents to the cheaper biosimilar alternative. Since 2018, the Norwegian pharmaceutical tendering has decided to only recommend the best offer among the reference agents and its biosimilars and considered the respective others as bioequivalent.

While iv b/tsDMARD generally provided better offers, they were almost consistently lower in proportion than sc/po b/tsDMARDs. The low interest in iv drugs compared to sc/po may be related to the iv drugs’ additional cost and less favorable patient satisfaction [24, 25]. In Norway, by our understanding, the overall cost of iv b/tsDMARD entails higher additional healthcare expenditure due to medical materials, training of anyone involved in administrating the iv b/tsDMARD, the cost of implementing a working staff to administrate the drug, and patient transportation (either out of pocket or governmentally paid). In Norway, in decentralized areas, some patients have to travel up to 200–300 km to their nearest rheumatologist. The iv rituximab reference is an exception to the aforementioned observation. Rituximab reference was either the highest or second highest in proportion in eight out of ten years for current users, five for non-naïve, and once for naïve, albeit it was the tender-winning drug only once. An important clarification is that rituximab was only offered as a second-line drug, albeit not exclusively as the only second-line option. Despite being offered on the premises as a backup drug and advised only under certain circumstances as a first-line drug [26], it still acquired 5% on average across the ten-year period among naïve users. Provided the use of rituximab was not contraindicated, a positive outcome was documented when treating seropositive RA with rituximab [26, 27], which can explain the small, yet unexpected, proportion among naïve b/tsDMARD users.

While it can be argued that the NHPT’s effort to manage the Norwegian tender system for costly pharmaceuticals resulted in a reduced b/tsDMARD cost, it is also likely that the introduction of biosimilars played a central role [16]. Since the biosimilars’ development and approval expenditure is lower than that of reference agents, biosimilars can be sold for a lower cost and consequently stir market competition. This may reduce the overall pharmaceutical expenditure for the payers in European Union countries and, in turn, increase the availability of medications and improve access to care [28–30]. Similarly, as illustrated in this study, the biosimilars’ introduction into the Norwegian pharmaceutical market may have influenced the overall b/tsDMARD proportion, especially the proportion of corresponding reference agents. Following the introduction of the first biosimilar in 2014, the iv infliximab CT-P13, the iv b/tsDMARDs were able to compete with the sc b/tsDMARDs despite iv’s less favorable patient satisfaction and higher cost [24, 25]. The iv b/tsDMARDs reached their proportion peak in 2015, where their usage surpassed the sc/po b/tsDMARDs and acquired 57% and 66% of the total b/tsDMARDs proportion for naïve and non-naïve users, respectively. Infliximab CT-P13’s favor started to wane in 2016 when the sc/po b/tsDMARDs’ gained a biosimilar of their own, the sc etanercept SB4. Yet, among non-naïve users, these two biosimilars accounted for 67% of the 1270 new biosimilar switches, the highest proportion of switches overall. While the sc etanercept SB4 gained a higher proportion, the iv infliximab CT-P13 kept the high competition active. In 2017 and 2018, the two biosimilars acquired more than 70% of the total b/tsDMARD proportion among naïve users.

Although the NHPT’s goal was to achieve the lowest possible b/tsDMARD cost, health outcomes were still prioritized. As such, the prescribing physicians were urged to be vigilant and careful when considering the NHPT’s recommendations, especially when dealing with novel b/tsDMARDs [31]. Under the NHPT’s recommendation (i.e., the Norwegian pharmaceutical tender system) between 2010 and 2019, the observed health outcomes among RA patients in Norway did not worsen [16]. In fact, the observed remission rate increased from 42 to 67% using DAS28 [16].

Pharmaceutical tendering can be cost-beneficial in the short term, as reported on multiple occasions [16, 32, 33]. However, unless the potential pitfalls of long-term pharmaceutical tendering are adequately handled, its high competition can also lead to unwanted impacts on patient healthcare quality, government budgets, pharmaceutical supply and capacity, novel pharmaceutical development, and sustainability of affordable prices [33–35]. In highly cost-reducing (i.e., highly competitive) pharmaceutical tendering, the offers required to compete can be detrimental for pharmaceutical companies and consequently result in a disinterest in providing any offer [33]. This can also be observed in the current study, where some companies decided not to give any offers, presumptively due to the high offers required to partake. If companies do not renew contracts when they expire, patient treatment options may be reduced. Furthermore, if multiple competitors withdraw, the remaining b/tsDMARDs may face little competition, potentially increasing costs [33]. Reduced competition can also impact pharmacies, resulting in supply instability [35]. Furthermore, if the tender-winning pharmaceutical company cannot supply as agreed, other companies that did not win the tender may not serve as backup suppliers, affecting patient treatment availability [35].

The NHPT is now addressing these potential concerns by considering establishing lower-end cost limits to prevent too low b/tsDMARDs costs. The NHPT has also implemented risk-distributing strategies to ensure better availability. For the risk distribution, NHPT recommends different b/tsDMARDs with the same bioequivalence for different geographical areas in Norway, e.g., infliximab SB2 (Flixabi®) and infliximab GP1111 (Zessly®) in 2020 and adalimumab SB5 (Imaraldi®) and adalimumab GP2017 (Hyrimoz®) in 2022.

The issue concerning confidentiality is an additional potential pitfall. Confidential offers allow pharmaceutical companies to give high offers without disclosing the information to other buyers (e.g., countries) [36]. Releasing these offers can result in mistrust between the pharmaceutical companies and the governing body purchasing the medications, leading to delays in the supply of known and novel drugs. Data release of pharmaceutical tender offers has occurred three times in Norway [37]. That said, pharmaceutical companies maintain market control by keeping the offers secret, benefiting while having the wiggle room to sell medications at high prices in each country without the other countries knowing about it [36]. In turn, the buyers have reduced autonomy as they are still bound to acquire essential medication for their region or nation on the pharmaceutical company’s premises [38].

There are several limitations to this study. Firstly, it is a cross-sectional study with no statistical assessment, only interpretations of descriptive data. Our findings reflect population-level trends rather than individual patient trajectories. While this approach may help understand broad patterns and associations, it may overlook individual differences in treatment responses and disease progression, which could provide more nuanced insights into the impact of Norway’s pharmaceutical tendering system on RA treatment patterns. Also, as a cross-sectional study, specific trends in change and order of b/tsDMARDs among non-naïve users are unknown. Longitudinal studies may provide more detailed insights into these aspects in the future.

In accordance with the national arthritis registry (NorArtritt) [39], which has cross-validated data with the Norwegian Patient Registry [40], data from our included centers demonstrates a comparable number of included RA patients and a similar b/tsDMARD treatment ratio.

Although our study’s external validity is comparable to that of NorArtritt, both our study and the NorArtritt registry only cover about 60% of the national RA population (2019 NorArtritt report) [39]. As a result, our study does not represent the entire demographic of RA patients and b/tsDMARD RA users in Norway, especially since some centers do not use or have only recently implemented the GTI system. As such, the increase in the patient population during our study period is more likely due to inclusion quantity registrations rather than due to an increase in the incidence of RA. In fact, there seems to be a decreasing trend in the incidence of RA [41]. This highlights the difficulty in interpreting trends in observational data and that it should be done cautiously. Despite these limitations, we believe our study has acceptable external validity and provides valuable, real-world insights into the use of b/tsDMARDs in Norway and how this usage may align with the national tender system.

An in-depth assessment of each drug from each center is not provided. The regional depot was also not accounted for, or the regional b/tsDMARD proportion. The geographical aspect also presents a challenge as there is a much longer travel time for RA patients in northern Norway compared to centralized areas like Bergen and Oslo. The impact of these geographical differences on treatment selection, especially the difference between decentralized and centralized areas based on iv vs. sc treatment, was not evaluated.

An additional potential limitation is our assumption that all patients receive their prescribed medication throughout the year. Given the design of our study, we used the most recent data entry from each year, leaving it unclear if a patient discontinued the drug during the year and the precise start date of treatment. Also, while all centers are expected to follow a standardized patient monitoring process, the reality of clinical practice often deviates from this standard due to various factors, e.g., doctors’ clinical decisions, logistical constraints, and practical implementation barriers. Disparities in data, as evidenced by missing data, reflect these complexities and the challenges of achieving uniform structured data collection across all centers. Lastly, while the highest and second highest proportions were outlined and presented as core elements of tendering being impactful, there were some cases where the difference between the second highest and the third highest proportion was only a few percentages apart.

The study also has multiple strengths. It is a unique study exploring the proportion of 13 different b/tsDMARDs across ten years, providing an overview of current, naïve and non-naïve b/tsDMARD users using real-life data from ten rheumatological outpatient clinics in Norway. It distinguishes between sc/po and iv b/tsDMARDs and between biosimilars and non-biosimilars b/tsDMARDs. Visual interpretation of the tender ranks compared with the proportion of the different user groups is also provided. To our awareness, this is the first study providing such a thorough assessment of b/tsDMARD tendering in Norway.

## Conclusion

The study’s observative finding of the pharmaceutical tendering process from 2010 to 2019 reveals a possible link between tendering outcomes and the use of b/tsDMARDs among various patient groups. According to the data, winning the tender appears to have an impact on b/tsDMARD usage in naive users and non-naive users and a less pronounced impact on current users. While this was a general observation taken from multiple annual tenders, sc/po b/tsDMARDs were generally favored over iv b/tsDMARDs despite higher-ranked offers for the iv b/tsDMARDs. These interpretations, however, are based on observed descriptions.

We also observed that the current b/tsDMARD proportion for several drugs remained high despite a relatively low ranking. This observation may be explained based on the “do not switch from a winning team” principle but also due to the lack of available data or recommendations to promote a cost-beneficial non-medical switch between b/tsDMARDs with different substances.

Building a solid market foundation appears to be an effective strategy for resisting proportion decrease when the necessary offers are not cost-effective for the individual pharmaceutical company. The foundation for some b/tsDMARDs, e.g., etanercept reference, was built prior to initial tendering resulting in a clear advantage. However, the dynamic between etanercept reference and its biosimilar etanercept SB4 is also a great example of how a solid foundation can be challenged and disrupted upon the introduction of corresponding biosimilars.

The pharmaceutical tender system implemented in Norway appears to favor the pharmaceutical company that provides a good offer by increasing their b/tsDMARD proportion among naïve and non-naïve users. Biosimilars contributed substantially to the competition by likely increasing market proportion.

### Supplementary Information

**Additional file 1: Supplementary Table 1.** Overview of excluded patients and registration errors within the BioRheuma data

## Data Availability

Data are available on reasonable request and must be approved by all participating centers. Please contact the corresponding author by email to request the data from this study.

## Abbreviations

ANOVA: One-way analysis of variance
anti-CCP: anti-cyclic citrullinated peptide
BE: Biosimilar equivalent
BMI: Body mass index
b/tsDMARD: biologic and targeted synthetic disease-modifying antirheumatic drugs
CRP: C-reactive protein
DAS28: Composite 28 joint count Disease Activity Score using CRP
ESR: Erythrocyte sedimentation rate
IGA: Investigator global assessment
iv: intravenous
NHPT: Norwegian hospital procurement trust
NO: No offer
PGA: Patient global assessment
po: per oral
PROM: Patient-reported outcome measures
RA: Rheumatoid arthritis
REC: Regional ethical committee
RF: Rheumatoid factor
sc: subcutaneous
sc-po: subcutaneous and per oral
SJC28: 28 swollen joint count
SPSS: Statistical package for social sciences
TNFi: Tumor necrosis factor inhibitors
TJC28: 28 tender joint count
VAS: Visual analog scale

## References

[1] Gabriel SE, Michaud K (2009). Epidemiological studies in incidence, prevalence, mortality, and comorbidity of the rheumatic diseases. Arthritis Res Ther.

[2] Put S, Westhovens R, Lahoutte T, Matthys P (2014). Molecular imaging of rheumatoid arthritis: emerging markers, tools, and techniques. Arthritis Res Ther.

[3] Whalley D, McKenna SP, de Jong Z, van der Heijde D (1997). Quality of life in rheumatoid arthritis. Br J Rheumatol.

[4] Zhou Y, Wang X, An Y, Zhang X, Han S, Li X (2018). Disability and health-related quality of life in chinese patients with rheumatoid arthritis: a cross-sectional study. Int J Rheum Dis.

[5] Hsieh P-H, Wu O, Geue C, McIntosh E, McInnes IB, Siebert S (2020). Economic burden of rheumatoid arthritis: a systematic review of literature in biologic era. Ann Rheum Dis.

[6] Boonen A, Severens JL (2011). The burden of illness of rheumatoid arthritis. Clin Rheumatol.

[7] Jansen JP, Buckley F, Dejonckheere F, Ogale S (2014). Comparative efficacy of biologics as monotherapy and in combination with methotrexate on patient reported outcomes (PROs) in rheumatoid arthritis patients with an inadequate response to conventional DMARDs–a systematic review and network meta-analysis. Health Qual Life Outcomes.

[8] Smolen JS, Landewé RBM, Bijlsma JWJ, Burmester GR, Dougados M, Kerschbaumer A (2020). EULAR recommendations for the management of rheumatoid arthritis with synthetic and biological disease-modifying antirheumatic drugs: 2019 update. Ann Rheum Dis.

[9] Aga AB, Lie E, Uhlig T, Olsen IC, Wierød A, Kalstad S (2015). Time trends in disease activity, response and remission rates in rheumatoid arthritis during the past decade: results from the NOR-DMARD study 2000–2010. Ann Rheum Dis.

[10] Sokka T, Pincus T (2009). Rheumatoid arthritis: strategy more important than agent. Lancet.

[11] Haugeberg G, Hansen IJ, Soldal DM, Sokka T (2015). Ten years of change in clinical disease status and treatment in rheumatoid arthritis: results based on standardized monitoring of patients in an ordinary outpatient clinic in southern Norway. Arthritis Res Ther.

[12] Putrik P, Ramiro S, Kvien TK, Sokka T, Pavlova M, Uhlig T (2014). Inequities in access to biologic and synthetic DMARDs across 46 european countries. Ann Rheum Dis.

[13] Bergstra SA, Branco JC, Vega-Morales D, Salomon-Escoto K, Govind N, Allaart CF (2018). Inequity in access to bDMARD care and how it influences disease outcomes across countries worldwide: results from the METEOR-registry. Ann Rheum Dis.

[14] Putrik P, Ramiro S, Kvien TK, Sokka T, Uhlig T, Boonen A (2014). Variations in criteria regulating treatment with reimbursed biologic DMARDs across european countries. Are differences related to country’s wealth?. Ann Rheum Dis.

[15] Sykehusinnkjøp. Pharmaceutical Strategy Of The Norwegian Hospital Procurement Trust (Sykehusinnkjøp HF) 2020 [Available from: https://sykehusinnkjop.no/pharmaceutical-strategy-of-the-norwegian-hospital-procurement-trust-sykehusinnkjop-hf.

[16] Brkic A, Diamantopoulos AP, Haavardsholm EA, Fevang BTS, Brekke LK, Loli L (2022). Exploring drug cost and disease outcome in rheumatoid arthritis patients treated with biologic and targeted synthetic DMARDs in Norway in 2010–2019 - a country with a national tender system for prescription of costly drugs. BMC Health Serv Res.

[17] van Gestel AM, Haagsma CJ, van Riel PL (1998). Validation of rheumatoid arthritis improvement criteria that include simplified joint counts. Arthritis Rheum.

[18] Stavem K (2021). Switching from one reference biological to another in stable patients for non-medical reasons: a literature search and brief review. J Mark Access Health Policy.

[19] Feagan BG, Marabani M, Wu JJ, Faccin F, Spronk C, Castañeda-Hernández G (2020). The Challenges of switching therapies in an evolving multiple Biosimilars Landscape: a narrative review of current evidence. Adv Ther.

[20] Jørgensen KK, Olsen IC, Goll GL, Lorentzen M, Bolstad N, Haavardsholm EA (2017). Switching from originator infliximab to biosimilar CT-P13 compared with maintained treatment with originator infliximab (NOR-SWITCH): a 52-week, randomised, double-blind, non-inferiority trial. The Lancet.

[21] Goll GL, Jørgensen KK, Sexton J, Olsen IC, Bolstad N, Haavardsholm EA (2019). Long-term efficacy and safety of biosimilar infliximab (CT‐P13) after switching from originator infliximab: open‐label extension of the NOR‐SWITCH trial. J Intern Med.

[22] Glintborg B, Loft AG, Omerovic E, Hendricks O, Linauskas A, Espesen J (2019). To switch or not to switch: results of a nationwide guideline of mandatory switching from originator to biosimilar etanercept. One-year treatment outcomes in 2061 patients with inflammatory arthritis from the DANBIO registry. Ann Rheum Dis.

[23] Glintborg B, Sørensen IJ, Loft AG, Lindegaard H, Linauskas A, Hendricks O (2017). A nationwide non-medical switch from originator infliximab to biosimilar CT-P13 in 802 patients with inflammatory arthritis: 1-year clinical outcomes from the DANBIO registry. Ann Rheum Dis.

[24] Anderson KC, Landgren O, Arend RC, Chou J, Jacobs IA (2019). Humanistic and economic impact of subcutaneous versus intravenous administration of oncology biologics. Future Oncol.

[25] Heald A, Bramham-Jones S, Davies M (2021). Comparing cost of intravenous infusion and subcutaneous biologics in COVID-19 pandemic care pathways for rheumatoid arthritis and inflammatory bowel disease: a brief UK stakeholder survey. Int J Clin Pract.

[26] Smolen JS, Landewé R, Breedveld FC, Buch M, Burmester G, Dougados M (2014). EULAR recommendations for the management of rheumatoid arthritis with synthetic and biological disease-modifying antirheumatic drugs: 2013 update. Ann Rheum Dis.

[27] Mok CC (2013). Rituximab for the treatment of rheumatoid arthritis: an update. Drug Des Devel Ther.

[28] Troein P, Newton M, Scott K (2020). The impact of biosimilar competition in Europe.

[29] Agency EM. Biosimilars in the EU - Information guide for healthcare professionals. 2019. https://www.ema.europa.eu/en/documents/leaflet/biosimilars-eu-information-guide-healthcare-professionals_en.pdf.

[30] Kvien TK, Patel K, Strand V (2022). The cost savings of biosimilars can help increase patient access and lift the financial burden of health care systems. Semin Arthritis Rheum.

[31] Association of the Pharmaceutical Industry in Norway. Public procurements in the pharmaceutical area - An important tool for good healthcare [Offentlige innkjøp på legemiddelområdet - Et viktig verktøy for god helsetjeneste]. 2015. https://www.lmi.no/download.php?file=/wp-content/uploads/2015/11/offentlige_innkj_p_p_legemiddelomr_det.pdf.

[32] Dylst P, Vulto A, Simoens S (2011). Tendering for outpatient prescription pharmaceuticals: what can be learned from current practices in Europe?. Health Policy.

[33] Kanavos P, Seeley L, Vandoros S. Tender systems for outpatient pharmaceuticals in the European Union: evidence from the Netherlands, Germany and Belgium. LSE Health London School of Economics. 2009. https://www.researchgate.net/publication/48910434_Tender_Systems_for_Outpatient_Pharmaceuticals_in_the_European_Union_Evidence_from_the_Netherlands_Germany_and_Belgium, https://www.politico.eu/wp-content/uploads/2019/02/Tender-systems-for-outpatient-pharmaceuticals-in-the-EU.pdf

[34] Dagens Medisin. Tender - for as long as it lasts [Anbud - så lenge det varer] 2022 [Available from: https://www.dagensmedisin.no/artikler/2022/02/11/anbud--sa-lenge-det-varer.

[35] Carradinha H (2009). Tendering short-term pricing policies and the impact on patients, governments and the sustainability of the generic medicines industry. J Generic Med.

[36] Tranvåg EJ. Confidential drug prices undermine trust in the system. Konfidensielle legemiddelpriser undergraver tilliten til systemet. Tidsskrift for den Norske laegeforening: tidsskrift for praktisk medicin, ny raekke. 2019;139(9). 10.4045/tidsskr.19.0284 31140256

[37] Anbud365. Embarrassing leakage of drug prices, Norwegian Hospital Procurement Trust accepts full blame [Pinlig lekkasje av legemiddel-priser, Sykehusinnkjøp la seg paddeflate] 2022 [cited 2022. Available from: https://www.anbud365.no/bransjer/helse-omsorg/pinlig-lekkasje-av-legemiddel-priser-sykehusinnkjop-la-seg-paddeflate/.

[38] Morgan SG, Vogler S, Wagner AK (2017). Payers’ experiences with confidential pharmaceutical price discounts: a survey of public and statutory health systems in North America, Europe, and Australasia. Health Policy.

[39] Registries] NSCfMQ. [Norwegian Arthritis Registry (NorArtritt)] 2023 [Available from: https://www.kvalitetsregistre.no/register/revmatologi/norsk-kvalitetsregister-artrittsykdommer-norartritt.

[40] Healthdata. Norwegian Patient Registry 2023 [Available from: https://helsedata.no/en/forvaltere/norwegian-directorate-of-health/norwegian-patient-registry-npr.

[41] Uhlig T, Kvien TK (2005). Is rheumatoid arthritis disappearing?. Ann Rheum Dis.

