# Supplementary material for: Exploring the impact of the national tender system on the use of costly drugs treating rheumatoid arthritis patients in ten rheumatology centers in Norway (2010–2019)
Source: BMC Health Serv Res. 2023 Sep 7;23:968. doi: 10.1186/s12913-023-09975-7 (PMC10486045; doi:10.1186/s12913-023-09975-7)
Supplement: Supplementary file 1 — Additional file 1: Supplementary Table 1. Overview of excluded patients and registration errors within the BioRheuma data [file 12913_2023_9975_MOESM1_ESM.docx]

**Supplementary Table 1:** Overview of excluded patients and registration errors within the BioRheuma data

|  | **2010** | **2011** | **2012** | **2013** | **2014** | **2015** | **2016** | **2017** | **2018** | **2019** |
| --- | --- | --- | --- | --- | --- | --- | --- | --- | --- | --- |
| **Registered BioRheuma patients** | | | | | | | | | | |
| Total registered BioRheuma patients | 4909 | 7256 | 7993 | 7278 | 8023 | 9057 | 9176 | 9225 | 9102 | 9335 |
| Included BioRheuma patients | 4885 (99.5%) | 7230 (99.6%) | 7970 (99.7%) | 7248 (99.6%) | 7993 (99.6%) | 9010 (99.5%) | 9037 (98.5%) | 9129 (99.0%) | 9048 (99.4%) | 9280 (99.4%) |
| Excluded BioRheuma patients | 24 (0.5%) | 26 (0.4%) | 23 (0.3%) | 30 (0.4%) | 30 (0.4%) | 47 (0.5%) | 139 (1.5%) | 96 (1.0%) | 54 (0.6%) | 55 (0.6%) |
| **Registration of the 13 b/tsDMARD** | | | | | | | | | | |
| Total registrations | 1934 | 2855 | 3134 | 3059 | 3418 | 3686 | 3770 | 3867 | 3867 | 4153 |
| b/tsDMARDs prescriptions | 1910 (98.8%) | 2829 (99.1%) | 3111 (99.3%) | 3029 (99.0%) | 3388 (99.1%) | 3639 (98.7%) | 3631 (96.3%) | 3771 (97.5%) | 3813 (98.6%) | 4098 (98.7%) |
| Registration Errors | 24 (1.2%) | 26 (0.9%) | 23 (0.7%) | 30 (1.0%) | 30 (0.9%) | 47 (1.3%) | 139 (3.7%) | 96 (2.5%) | 54 (1.4%) | 55 (1.3%) |
| **Overview of excluded registration errors** | | | | | | | | | | |
| In bDMARDs and tsDMARDs | 0 | 0 | 0 | 0 | 0 | 0 | 0 | 4 | 24 | 16 |
| In tsDMARDs | 0 | 0 | 0 | 0 | 0 | 0 | 0 | 0 | 2 | 4 |
| In bDMARDs | 10 | 10 | 6 | 5 | 13 | 14 | 16 | 9 | 10 | 2 |
| In TNFi | 11 | 13 | 17 | 23 | 13 | 30 | 119 | 79 | 21 | 18 |
| In non-TNFi | 4 | 4 | 1 | 2 | 4 | 3 | 4 | 5 | 0 | 16 |
| Overlapping registration errors | 1 | 1 | 1 | 0 | 0 | 0 | 0 | 1 | 3 | 1 |

*Abbreviations*: **b/tsDMARDs** = biologic and target synthetic Disease-Modifying Antirheumatic Drugs. **bDMARDs** = biological DMARDs. **tsDMARDs** = target synthetic DMARDs. **TNFi** = Tumor Necrosis Factor Inhibitor**.**
